# Integrated ATAC-seq and RNA-seq Analysis of In Vitro Cultured Skeletal Muscle Satellite Cells to Understand Changes in Cell Proliferation

**DOI:** 10.3390/cells13121031

**Published:** 2024-06-13

**Authors:** Zeyu Ren, Siyi Zhang, Liangyu Shi, Ao Zhou, Xin Lin, Jing Zhang, Xiusheng Zhu, Lei Huang, Kui Li

**Affiliations:** 1 Hubei Key Laboratory of Animal Nutrition and Feed Science, Laboratory of Genetic Breeding, Reproduction and Precision Livestock Farming, Hubei Provincial Center of Technology Innovation for Domestic Animal Breeding, Wuhan Polytechnic University, Wuhan 430023, China; r17340512105@126.com (Z.R.); m15007191047@126.com (S.Z.); liangyu_shi@whpu.edu.cn (L.S.); zhouao2008@aliyun.com (A.Z.); 2 Shenzhen Branch, Guangdong Laboratory of Lingnan Modern Agriculture, Key Laboratory of Livestock and Poultry Multi-omics of MARA, Agricultural Genomics Institute at Shenzhen, Chinese Academy of Agricultural Sciences, Shenzhen 518124, China; likui@caas.cn; 3 College of Animal Science and Veterinary Medicine, Tianjin Agricultural University, Tianjin 300392, China; linxin000325@163.com

**Keywords:** pig, satellite cells, cell proliferation, ATAC-seq

## Abstract

Skeletal muscle satellite cells, the resident stem cells in pig skeletal muscle, undergo proliferation and differentiation to enable muscle tissue repair. The proliferative and differentiative abilities of these cells gradually decrease during in vitro cultivation as the cell passage number increases. Despite extensive research, the precise molecular mechanisms that regulate this process are not fully understood. To bridge this knowledge gap, we conducted transcriptomic analysis of skeletal muscle satellite cells during in vitro cultivation to quantify passage number-dependent changes in the expression of genes associated with proliferation. Additionally, we explored the relationships between gene transcriptional activity and chromatin accessibility using transposase-accessible chromatin sequencing. This revealed the closure of numerous open chromatin regions, which were primarily located in intergenic regions, as the cell passage number increased. Integrated analysis of the transcriptomic and epigenomic data demonstrated a weak correlation between gene transcriptional activity and chromatin openness in expressed genic regions; although some genes (e.g., *GNB4* and *FGD5*) showed consistent relationships between gene expression and chromatin openness, a substantial number of differentially expressed genes had no clear association with chromatin openness in expressed genic regions. The p53-p21-RB signaling pathway may play a critical regulatory role in cell proliferation processes. The combined transcriptomic and epigenomic approach taken here provided key insights into changes in gene expression and chromatin openness during in vitro cultivation of skeletal muscle satellite cells. These findings enhance our understanding of the intricate mechanisms underlying the decline in cellular proliferation capacity in cultured cells.

## 1. Introduction

The pig (*Sus scrofa*) is an economically valuable agricultural animal that serves as a major global source of meat [1]. In part due to their high growth rate, pigs are an appealing model species for genetic muscle diseases, giving them further value in the biomedical field [2,3]. Skeletal muscle constitutes the largest proportion of all bodily tissues and has vital roles in voluntary movement in addition to metabolic and endocrine functions [4,5]. The connective tissues and muscle fibers give skeletal muscle strong contractile and relaxation abilities that generate movement [6,7]. Furthermore, skeletal muscle has remarkable capacities for regeneration and self-repair after injury; this process primarily relies on skeletal muscle satellite cells [8,9].

Satellite cells are the resident stem cells within skeletal muscle. They perform essential functions during postnatal development, skeletal muscle hypertrophy prior to maturity, and skeletal muscle regeneration throughout the lifespan [10,11]. In the absence of external stimulation, satellite cells typically remain dormant due to transient inhibition of the cell cycle. After muscle injury of any severity, ranging from minor strain to severe trauma, signals originating from the impacted microenvironment activate satellite cells, triggering myogenic capacity, proliferation, and subsequent differentiation into myoblasts [12,13]. This process of activation, proliferation, differentiation, and fusion gives rise to multi-nucleated cells called myotubes that have similar characteristics to the muscle fibers from which they originate [14].

Cultured meat production requires the large-scale cultivation of functional stem cells in vitro. However, maintenance of pig muscle stem cell functionality during extended periods of in vitro culture has proven challenging; pig muscle stem cells demonstrate reduced myotube formation and regenerative capabilities following long-term culture [15]. Satellite cells cease to grow almost completely after in vitro passaging to the 12th passage, and most cells exhibit signs of nuclear condensation (a characteristic of cellular senescence) and a reduced proliferative capacity. Cellular senescence refers to a persistent cell cycle arrest state in which aging cells cease to divide during the G1 or G2/M phase. This arrest is induced by various cell cycle inhibitory factors such as p53, p21, p16INK4a, and p15INK4b [5]. During the senescence process, cells that ultimately succumb to multifactorial stress undergo widespread changes in histone disassembly, accompanied by local and global chromatin remodeling, imbalances in activating and inhibitory histone modifications, and overall transcriptional alterations [16]. Skeletal muscle satellite cells that have undergone in vitro expansion for more than five passages display significantly lower differentiation potential and lose their ability to repair muscle damage in vivo [7,17].

Although previous studies have suggested that in vitro culturing of skeletal muscle satellite cells leads to a loss of stem cell characteristics over time [15,17], limited research has been conducted to identify the associated changes in gene expression patterns and chromatin accessibility. To address this knowledge gap, we cultured primary satellite cells isolated from newborn Duroc pig skeletal muscle in vitro for three, five, and seven passages. Transposase-accessible chromatin sequencing (ATAC-seq) and RNA sequencing (RNA-seq) analyses were then conducted to quantify changes in gene expression and chromatin accessibility in these cells. This approach was designed to identify key genes involved in the loss of stem cell characteristics during in vitro culturing of porcine skeletal muscle cells, providing key targets for future studies.

## 2. Materials and Methods

### 2.1. Cell Culture

We procured first-passage skeletal muscle satellite cells derived from newborn Landrace pigs and cultured them in vitro as F1 passage cells from iCell Bioscience Inc. (located in Shanghai, China) at a density of 500,000 cells per flask in T25 culture flasks. Upon achieving 70% confluency, subculturing was carried out at a 1:3 ratio. The cell culture medium utilized in this study was sourced from iCell Bioscience Inc. (Shanghai, China) and comprised a basal medium for primary skeletal muscle cells, fetal bovine serum (FBS), antibiotics (penicillin/streptomycin, P/S), and a supplement specifically designed for primary skeletal muscle cell culture. The primary skeletal muscle cell culture supplement further contained both essential and non-essential amino acids, vitamins, hormones, growth factors, trace elements, as well as various organic and inorganic compounds. To prepare the culture medium, we introduced 10% FBS, 1% antibiotics, and 1% culture supplement into the basal medium. Cells were maintained in an incubator at 37 °C with 5% CO_2_.

### 2.2. Cell Differentiation

Porcine skeletal muscle satellite cells from the 3rd, 5th, and 7th generations were separately revived and cultured in 6-well plates. Upon reaching a cell density of 70–80%, the differentiation medium supplemented with 2% horse serum (16050122, Invitrogen, Carlsbad, CA, USA) was substituted to induce myogenic differentiation. The differentiation medium was refreshed every 24 h, and cellular morphological changes were examined under a microscope after a 3-day induction period.

### 2.3. Immunofluorescent Staining

To verify the purity and differentiation potential of skeletal muscle satellite cells, we performed immunofluorescence staining experiments for Desmin, MyoD1, and MF20 on F3, F5, and F7 passages of skeletal muscle satellite cells. The cells were cultured in a 6-well plate at a density of 50,000 cells per well. When the cells reached 50–60% confluency, they were washed twice with PBS and fixed with 4% paraformaldehyde (P0099, Beyotime, Shanghai, China) at room temperature for 30 min. After washing twice with PBS, the cells were incubated with 0.2% Triton X-100 (V900502, Sigma-Aldrich, St. Louis, MO, USA) at room temperature for 20 mins. Following another two washes with PBS, the cells were incubated with 2% BSA at room temperature for 1 h. We used MyoD1 Rabbit pAb (A0671, ABclonal, Wuhan, China), Desmin (GTX103557, Genetex, San Antonio, TX, USA) and Myosin 4 Monoclonal Antibody (MF20) (14-6503-82, Invitrogen) antibodies. The primary antibodies were diluted in 2% BSA to an appropriate ratio and added to the cells, which were then incubated overnight at 4 °C. After washing twice with PBS, the cells were incubated with the secondary antibody diluted in 2% BSA at room temperature in the dark for 1 h. Anti-rabbit IgG (4412s, CST) was used as the secondary antibody for desmin and MyoD, and anti-mouse IgG (4409S, CST) was used as the secondary antibody for MF20. The negative control group was established by omitting the addition of primary antibody. Following another two washes with PBS for 5 min each, the cells were stained with DAPI staining solution (C0065, Solarbio, Beijing, China) at room temperature in the dark for 5 min. After washing twice with PBS, an appropriate amount of PBS was added, and the cells were imaged using a confocal fluorescence microscope (Nikon A1HD25, Tokyo, Japan).

### 2.4. Flow Cytometry

Skeletal muscle satellite cells were cultured and preserved after three, five, and seven passages. These cells were simultaneously resuscitated for further experiments. After reaching a confluence rate of 50–70%, the cells were digested with 1 mL trypsin for 2 min, then centrifuged at 1000× *g* rpm for 3 mins. The resulting cells were resuspended through dropwise addition of cold (−20 °C) 75% ethanol before incubation overnight at −20 °C. Cells were then collected via centrifugation at 1000× *g* rpm for 3 mins and resuspended in 500 μL of PI/RNase Staining Buffer (BD Biosciences, Franklin Lake, NJ, USA). Cells were incubated for 15 min at room temperature in a light-protected environment, then collected via centrifugation. Finally, cells were resuspended in phosphate-buffered saline (PBS) and analyzed with flow cytometry (Beckman Coulter, Brea, CA, USA, CytoFLEX) within 1 h. Flow cytometry results were analyzed and graphed using Flow Jo 10.9.0 software. Differences in cell cycle states between passages were assessed with one-way analysis of variance (ANOVA).

### 2.5. Cell Proliferation Assay

Cell proliferation was assessed using the Cell-Light EdU Apollo 488 In Vitro Kit (RIBOBIO, Guangzhou, China), which uses 5-ethynyl-2′-deoxyuridine (EdU). Resuscitated cells from the third, fifth, and seventh passages were cultured to the logarithmic growth stage, then digested and counted. Cells were aliquoted into 12-well plates at a density of 50,000 cells per well (with two replicate wells per passage) and cultured to the normal growth stage. EdU, diluted 1:5000 in cell complete medium, was added to each well and the plate was incubated at 37 °C for 2 h. Cells were fixed in PBS containing 4% paraformaldehyde, then sequentially incubated with 1 mL 1× Apollo staining solution and 1 mL 1× Hoechst 33,342 reaction solution. After rinsing with PBS, cells were visually inspected and images were captured with an inverted fluorescence microscope (Nikon, Ts2). Images were quantified with ImageJ software [18]. Differences in cell proliferation capacity between passages were assessed with one-way ANOVA.

### 2.6. RNA Extraction and Quantitative Reverse Transcription PCR (qRT-PCR)

To validate the results of the RNA-seq experiment, we conducted qRT-PCR experiments. Total RNA was extracted with TRIzol reagent (Omega Bio-Tek, Norcross, GA, USA). RNA sample quality was evaluated with a Qubit fluorometer (Thermo Fisher Scientific, Waltham, MA, USA) and a NanoDrop 2000 (NanoDrop Technologies, Wilmington, DE, USA). First-strand cDNA was synthesized using the RevertAid First Strand cDNA Synthesis Kit (Thermo Fisher Scientific, Waltham, MA, USA). Primers (Table S1) were designed with Oligo7. qRT-PCR reactions were conducted with Taq Pro Universal SYBR qPCR Master Mix (Vazyme, Nanjing, China) on a CFXOpus96 instrument (Bio-Rad, Hercules, CA, USA) with the following reaction conditions: 95 °C for 30 s; 40 cycles of 95 °C for 10 s; and 60 °C for 30 s. The melting curve was plotted at a temperature 2 degrees lower than the annealing temperature at a rate of 0.5 °C per min until 90 °C was reached. All of the cycle threshold values were then collected. Gene expression values were normalized to the internal control gene *GAPDH* [19] with the 2^−ΔΔCt^ method [20].

### 2.7. mRNA-seq Library Construction, Sequencing, and Analysis

Total RNA was extracted as described above and evaluated with a Qubit fluorometer (Thermo Fisher Scientific, Waltham, MA, USA), a NanoDrop 2000 (NanoDrop Technologies, Wilmington, DE, USA), and an Agilent 2100 Bioanalyzer (Agilent Technologies, Santa Clara, CA, USA). A total of 1 μg RNA per sample was used. mRNA was isolated with VANTS mRNA Capture Beads (Vazyme, Nanjing, China), then fragmented. Fragments of 200–300 bp in length were selected for library construction with the VANTS Universal V8 RNA-seq Library Prep Kit for Illumina (Vazyme, Nanjing, China). Library quality was assessed with a Qubit fluorometer (Thermo Fisher Scientific, Waltham, MA, USA) and an Agilent 2100 Bioanalyzer (Agilent Technologies, Santa Clara, CA, USA). Sequencing was conducted by Novogene (Beijing, China).

Raw reads were trimmed to remove those of low quality using trim_galore (v0.6.7) [21]. The clean reads were mapped to the *S. scrofa* v11.1 reference genome (susScr11) [8] with STAR v2.7.10b [22]. SAMTools v1.6 was used to remove unmapped reads [23]. Significantly differentially expressed genes (DEGs) were called with the ‘DESeq2’ R package (v1.36.0) [24] using the following criteria: |log_2_(fold change)| ≥ 1 and false discovery rate (FDR)-adjusted *p* < 0.05. Genes were clustered based on expression trends with the Mffuz package [8].

### 2.8. ATAC-seq Library Construction, Sequencing, and Analysis

The Chromatin Profile Kit for Illumina (Novoprotein, SuZhou, China) was used to construct an ATAC-seq library for porcine skeletal muscle satellite cells following the manufacturer’s instructions. Briefly, cell nuclei were extracted and resuspended in Tn5 transposase reaction mix. After incubation for 30 min at 37 °C, fragment products were extracted with Tagment DNA Extract Beads. The PCR amplification reaction was carried out and the libraries were purified with DNA Clean Beads. Library quality was assessed using a Qubit fluorometer (Thermo Fisher Scientific, Waltham, MA, USA) and an Agilent 2100 Bioanalyzer (Agilent Technologies, Santa Clara, CA, USA). Sequencing was conducted by Novogene (Beijing, China).

Raw reads were trimmed with trim_galore v0.6.7 to eliminate low-quality reads [21]. The clean reads were mapped to the susScr11 reference genome using Bowtie2 v2.2.5 [25]. Unmapped reads, mitochondrial DNA, and duplicate reads were removed with SAMTools (v1.6) [23] and gatk4 (v4.4.0.0) [26] to yield a set of unique mapped reads. BAM files were converted to BigWig format with DeepTools (v3.5.1) [27] for visualization in the Integrative Genomics Viewer (IGV) (v.2.7.0) [28]. Accessible regions (peaks) for each replicate were identified in MACS2 v2.2.6 with following parameters: ‘--nomodel --shift -75 --extsize 150 -B --SPMR --keep-dup all --call-summits’ [29]. The threshold for a region to be called as a peak was Q < 0.05 [30]. Replicate sample similarity was assessed with the Irreproducibility Discovery Rate (IDR) (v2.0.4.2) [31], and peaks from replicate samples were merged in BEDTools (v2.30.0) [32]. The ‘ChIPseeker’ R package (v1.5.1) was used to assess peak distribution across various genomic regions [33]. Differential peaks (i.e., differentially accessible chromatin regions (DARs)) between cell passages were called with DiffBind v3.6.5 using the criteria |log2(fold change)| ≥ 1 and FDR-adjusted *p* < 0.05 [34].

### 2.9. Annotation and Functional Enrichment Analyses

Transcription factor binding motifs in chromatin peak regions were identified with the HOMER findMotifsGenome.pl tool at a cutoff of *p* < 0.05 [35]. Functional enrichment was assessed in DEGs and DARs using Gene Ontology (GO) and Kyoto Encyclopedia of Genes and Genomes (KEGG) annotations. Both analyses were conducted using the ‘clusterProfiler’ R package (v4.4.4) [36].

### 2.10. Statistical Analysis

Statistical analysis and graphing were performed using GraphPad Prism 9.0.0 software. One-way analysis of variance (ANOVA) was employed to analyze the differences between groups. Data are shown as the mean ± standard error of the mean (SD). The significance analysis of differences was as follows: *p* > 0.05 indicated no significant difference (ns); *p* < 0.05 indicated a significant difference (*); *p* < 0.01 indicated a highly significant difference (**); and *p* < 0.001 indicated an extremely significant difference (***).

## 3. Results

### 3.1. Identification of Skeletal Muscle Satellite Cells at Different Passages

**Figure 1:**
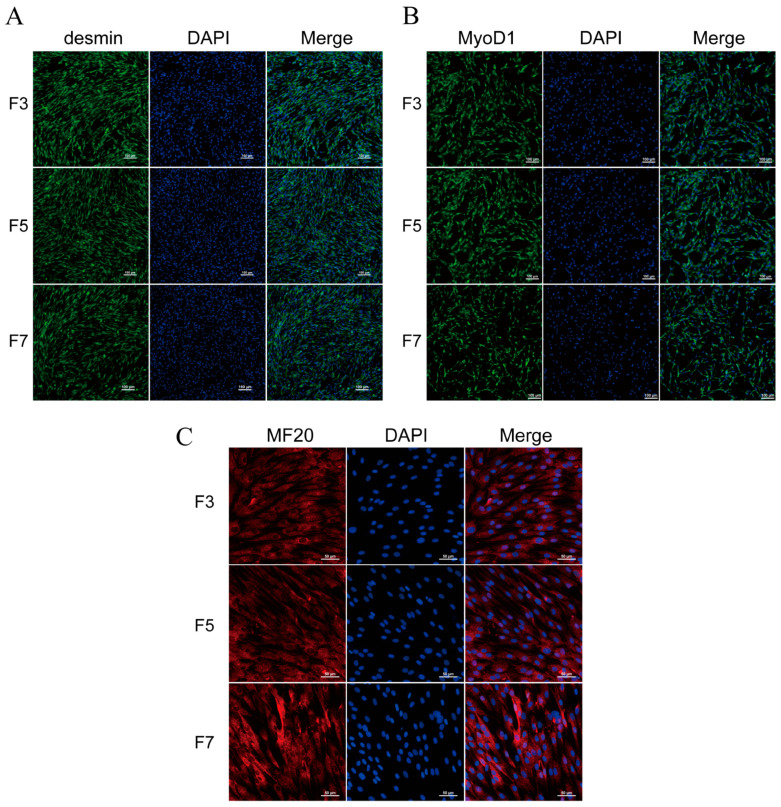
Immunofluorescence staining of skeletal muscle satellite cells. (**A**) Desmin and (**B**) MyoD1 immunofluorescence staining on F3, F5, and F7 passages of skeletal muscle cells, using a magnification of 200×. (**C**) MF20 immunofluorescence staining on F3, F5, and F7 passages of skeletal muscle cells after three days of differentiation, using a magnification of 400×.

To confirm the purity of porcine skeletal muscle satellite cells at different passages during in vitro culture and assess their stem cell characteristics, we performed immunofluorescent staining for Desmin and MyoD1 on F3, F5, and F7 cell populations. The results indicated that the rates of desmin-positive cells in F3, F5, and F7 passages were all above 90.2%. Similarly, the rates of myod1-positive cells were all above 92.3% (Figure 1A,B).

In order to evaluate the in vitro myogenic differentiation capability of porcine skeletal muscle satellite cells, we employed a differentiation medium supplemented with 2% horse serum to induce myogenesis. After 3 days of induction, immunofluorescence staining with the MF20 antibody was conducted. The results demonstrated the presence of MF20 in the differentiated cells of the F3, F5, and F7 passages (Figure 1C), while it remained absent in cells undergoing normal proliferation (Figure S1A). To ensure the experimental accuracy, a negative control method devoid of the primary antibody was employed (Figure S1B).

These findings demonstrate that porcine skeletal muscle satellite cells exhibit myogenic characteristics and are capable of undergoing myogenic differentiation during in vitro cultivation.

### 3.2. Weakened Proliferative Capacity in High-Passage Skeletal Muscle Satellite Cells

**Figure 2:**
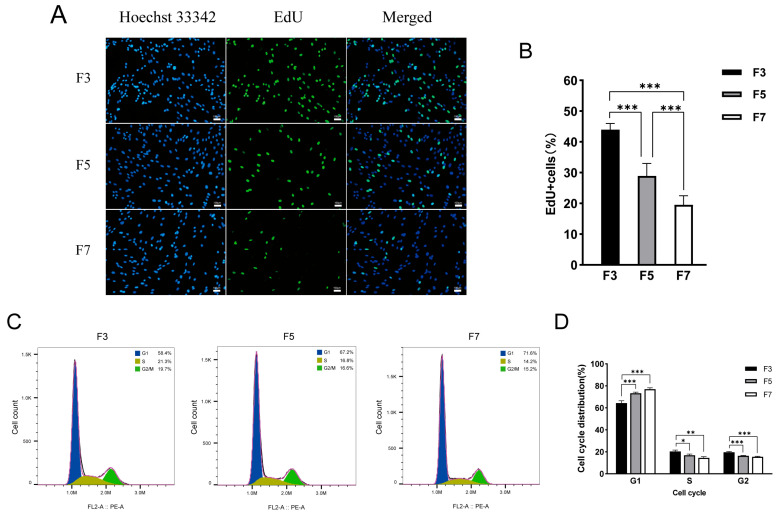
The cell proliferation potential and distribution of cell cycle phases of skeletal muscle satellite cells cultured in vitro vary across different passages. (**A**) Images of third-passage (F3), fifth-passage (F5), and seventh-passage (F7) pig skeletal muscle cells stained with 5-ethynyl-2′-deoxyuridine (EdU). Images were captured at 100× magnification. (**B**) Statistical analysis of the EdU positivity rate in cells from each passage. Data are displayed as mean ± SD, n = 9 independent experiments. *** *p* < 0.001. (**C**) Distribution of cell cycle phases among F3 (left), F5 (middle), and F7 (right) cells. (**D**) Quantification of the cell cycle phase distribution for each passage. Data are displayed as mean ± SD, n = 3 independent experiments. * *p* < 0.05, ** *p* < 0.01, *** *p* < 0.001.

In order to better understand the cellular physiological changes resulting in the in vitro passage that eventually led to the loss of proliferative potential, we first assessed the proliferative capacity of cells from different passages. Cell proliferation was quantified in each passage with EdU staining (Figure 2A). The number of EdU-positive cells gradually decreased from F3 to F7 samples, demonstrating a decline in the proliferative capacity of Landrace skeletal muscle satellite cells during in vitro culturing (Figure 2B). Furthermore, cell cycle analysis via flow cytometry revealed a notable rise in the number of cells in the G1 phase with each successive passage, corresponding to significant decreases in the populations of S-phase and G2-phase cells (Figure 2C,D). These results indicate that cell proliferation capacity is weakened during in vitro culturing, and the cell cycle is arrested in the G1 phase.

### 3.3. DEGs Were Enriched in Proliferation Functions

In order to identify genes with differential expression between high- and low-passage cells, high-throughput mRNA-seq was next conducted to compare gene expression levels between passages of cells. Sequencing generated a total of ~35–65 million clean reads per sample, corresponding to ~32–59 million reads per sample that could be mapped to the reference genome (Table S2). Correlation analysis (Figure S2A) and principal component analysis (PCA) (Figure S2B) were performed on all nine samples (comprising three replicates each of F3, F5, and F7 cells). There were strong correlations between replicate samples for each passage, indicating high replicability.

**Figure 3:**
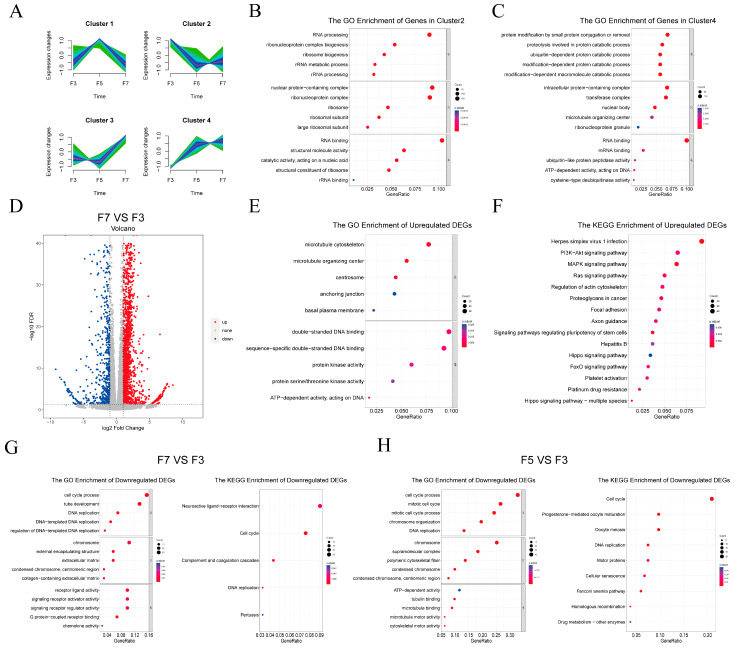
Functional analysis of differentially expressed genes across cell passages. (**A**) Gene clustering based on expression levels across passages. The x-axis represents the samples, while the y-axis represents centralized and normalized expression values. The mean expression trends of the genes within each cluster are depicted by the purple lines. (**B**,**C**) Gene Ontology (GO) enrichment analysis of the genes in cluster 2 (**B**) and cluster 4 (**C**). (**D**) Fold-change values for each gene in F7 compared to F3 cells. Gray points were not statistically significantly different between samples; red and blue points were significantly upregulated and downregulated, respectively, in F7 compared to F3 cells. Differences were considered statistically significant at |log2(fold change) ≥ 1| and false discovery rate-adjusted *p* < 0.05. (**E**,**F**) Functional enrichment analyses of upregulated DEGs in F7 vs. F3 group with (**E**) Gene Ontology (GO) annotations and (**F**) Kyoto Encyclopedia of Genes and Genomes (KEGG) biochemical pathway annotations. (**G**) Functional enrichment analyses of downregulated DEGs in F3 vs. F7 group with GO annotations and KEGG biochemical pathway annotations. (**H**) Functional enrichment analyses of downregulated DEGs in F5 vs. F3 group with GO annotations and KEGG biochemical pathway annotations.

To understand general trends in gene expression, genes were clustered based on expression levels across passages, revealing four distinct expression trends (Figure 3A). Genes in each cluster were then analyzed for functional enrichment. Genes in Cluster 1 were upregulated in F5 but downregulated in F7 compared to F3 cells. This cluster showed enrichment of the annotations “cell adhesion”, “signaling receptor activator activity”, and “cytokine activity” (Figure S2C). Cluster 2 contained genes that were downregulated in both F5 and F7 compared to F3 cells, and was primarily enriched in the terms “RNA processing”, “rRNA metabolic process”, and “RNA binding” (Figure 3B). Genes in Cluster 3 were downregulated in F5 but upregulated in F7 compared to F3 cells and were enriched in the annotations “cytoskeletal protein binding”, “actin binding”, and “GTPase activator activity” (Figure S2D). Genes in Cluster 4 were upregulated in both F5 and F7 compared to F3 cells and were enriched in the terms “ATP-dependent activity acting on DNA”, “proteolysis involved in protein catabolic process”, and “protein catabolic process modification-dependent” (Figure 3C).

To identify specific genes that may have been related to the decreased proliferative capacity of high-passage pig skeletal satellite cells, DEGs were identified between the passages. There were a total of 2,847 DEGs in F7 compared to F3 cells, comprising 2171 upregulated and 676 downregulated genes (Figure 3D). The comparison of F5 to F3 cells yielded slightly fewer DEGs at 2531, comprising 2190 upregulated and 341 downregulated DEGs (Figure S2E). GO annotation and KEGG biochemical pathway enrichment analyses were next conducted to assess the putative functions associated with the DEGs (Figure 3E–H and Figure S2F,G). Genes that were upregulated in F7 compared to F3 cells were enriched in GO annotations including “microtubule cytoskeleton”, “protein kinase activity”, and “ATP-dependent activity” (Figure 3E) and in several KEGG pathways related to cell proliferation and apoptosis such as the PI3K-Akt, MAPK, Ras, Hippo and p53 signaling pathways (Figure 3F). Genes upregulated in F5 compared to F3 cells were enriched in GO terms including “modification-dependent, ubiquitin-dependent protein catabolic process” and “ATP-dependent activity” (Figure S2F). These genes were also enriched in KEGG pathways related to cell proliferation, such as the MAPK and Ras signaling pathways and the actin cytoskeleton regulatory pathway (Figure S2G). Genes that were downregulated in F7 and/or F5 compared to F3 cells were significantly enriched in the GO term “cell cycle process” and in DNA replication signaling pathways (Figure 3G,H).

### 3.4. Dynamics of Chromatin Accessibility across Passages

To determine whether changes in gene expression levels were caused by alterations in chromatin accessibility, ATAC-seq was conducted in satellite cells from the F3, F5, and F7 passages. This produced ~52–136 million raw reads per sample, corresponding to ~36–97 million clean reads per sample that could be uniquely mapped to the reference genome (Table S3). Library quality was assessed through analysis of the insert length, which showed the expected distribution across all samples. The average fragment sizes were approximately 100 and 200 bp, suggesting the presence of nucleosome-free and mono-nucleosome-bound fragments. Chromatin was therefore determined to have been accessible to Tn5 transposase in all samples, consistent with the identified integer multiples of nucleosomes (Figure S3A).

**Figure 4:**
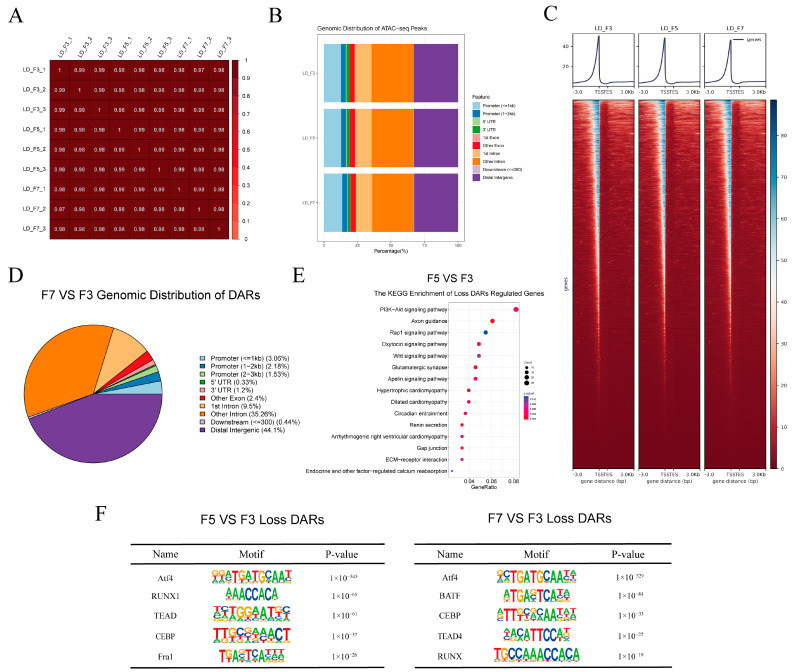
Differentially accessible chromatin regions (DARs) in seventh-passage (F7) and fifth-passage (F5) compared to third-passage (F3) cells. (**A**) Pairwise Pearson’s correlation for all nine samples (three replicates for each of the F3, F5, and F7 passages). (**B**) Genomic distribution of open chromatin peaks in each passage of cells. Functional regions were categorized as follows: promoter (≤1 kb), promoter (1–2 kb), 3′ untranslated region (UTR), 5′ UTR, first exon, other exon, first intron, other intron, downstream (≤300 Kb), and distal intergenic region. (**C**) Visualization of open chromatin signals within a 3 kb window surrounding the peak center. TSS, transcription start site; TES, transcription end site. (**D**) Genomic distribution of DARs in F7 vs. F3 gruop. (**E**) Kyoto Encyclopedia of Genes and Genomes (KEGG) classification of genes with decreased chromatin accessibility in F5 vs. F3 gruop. (**F**) Enriched transcription factor binding motifs in F5 and F7 compared to F3 cells.

IDR was next used to evaluate replicability by comparing replicate samples (comprising three per passage) (Figure S3B). Pairwise Pearson correlation coefficients between each pair of samples showed strong similarities between replicate samples and extensive differences between cells from different passages (Figure 4A). Further analyses were therefore conducted using a single combined dataset for each passage. The resulting average chromatin peaks were annotated using the reference genome, revealing similar genomic distribution patterns between cells of different passages (Figure 4B). Read mapping demonstrated significant enrichment of accessible regions within 3 kb of transcription start sites (TSSs) (Figure 4C). This was consistent with previously published ATAC-seq datasets [37], demonstrating the high quality of the ATAC-seq data.

DARs were identified by comparing peak locations across passages. In F7 compared to F3 cells, there were only 149 regions of more accessible chromatin (gain-DARs) and 767 regions of less accessible chromatin (loss-DARs). In F5 compared to F3 cells, there were only 198 gain-DARs and 1,487 loss DARs. Peak annotation analysis revealed that a majority of DARs were situated in distal intergenic regions (Figure 4D and Figure S3C). Further annotation of the DARs revealed 114 upregulated and 609 downregulated genes in DARs among F7 compared to F3 cells and 188 upregulated and 1,211 downregulated genes within DARs among F5 compared to F3 cells. Genes in loss-DARs among F5 compared to F3 cells were enriched in the PI3K-Akt, Rap1, and Wnt signaling pathways (Figure 4E).

We utilized the HOMER package to identify transcription factor binding motifs, and subsequently, the top five motifs significantly enriched in loss-associated DARs were presented. Notably, Atf4 binding sites were significantly enriched in loss-DARs in both F5 and F7 compared to F3 cells (Figure 4F). To further analyze the regions bound by Atf4, we divided the loss-DARs based on their genomic positions into three groups: promoter, distal intergenic, and intron regions. This allowed us to investigate the enrichment of Atf4 motif sequences on DARs in each respective region. We observed a significant enrichment of Atf4 binding sites within the distal intergenic DARs in both F5 and F7 compared to F3 cells (Figure S3D). Interestingly, DARs located in both the promoter and intron regions displayed a remarkable enrichment of Atf4 binding sites among F7 compared to F3 cells (Figure S3E).

### 3.5. Relationships between Chromatin Accessibility and Gene Expression across Passages

**Figure 5:**
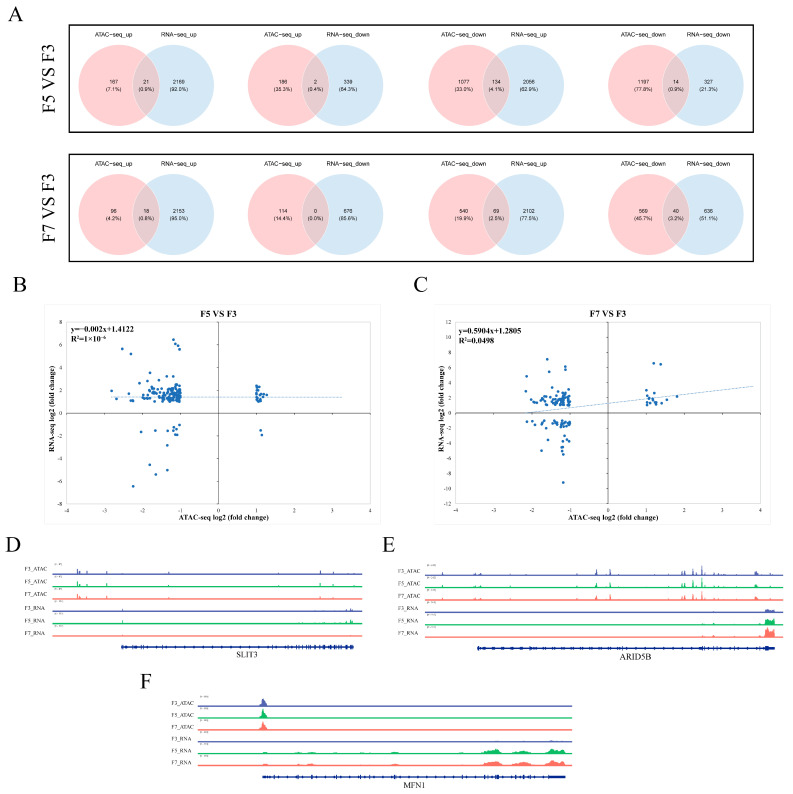
Integrated epigenomic and transcriptomic analysis. (**A**) Overlap between significantly differentially expressed genes (DEGs) and differentially accessible chromatin regions (DARs). ATAC-seq_down, genes that were less accessible in fifth- or seventh-passage (F5 and F7, respectively) cells compared to third-passage (F3) cells; ATAC-seq_up, genes that were more accessible in F5 or F7 than F3 cells; RNA-seq_up, DEGs that were upregulated in F5 or F7 compared to F3 cells; RNA-seq_down, DEGs that were downregulated in F5 or F7 compared to F3 cells. (**B**,**C**) Correlations between DEGs and genes within DARs in (**B**) F5 and (**C**) F7 compared to F3 cells. (**D**–**F**) Integrative Genomics Viewer snapshots showing chromatin peaks and RNA sequencing read levels for specific regions of (**D**) *SLIT3*, (**E**) *ARID5B*, and (**F**) *MFN1*. All three genes were differentially expressed in F5 and/or F7 compared to F3 cells, but showed no significant differences in chromatin accessibility.

To explore potential associations between chromatin accessibility and gene expression during cell culturing, an integrated transcriptomic–epigenomic analysis was performed. In F7 and F5 compared to F3 cells, there were a total of 21 and 18 upregulated genes within gain-DARs. Interestingly, in the F5 and F7 compared to F3 groups, 134 and 69 upregulated DEGs were present in loss-DARs (Figure 5A). In total, 1399 genes showed differential accessibility between F5 and F3 samples, 496 of which were expressed at extremely low or undetectable levels across all passages. Similarly, 723 genes were differentially accessible in F7 compared to F3 cells, 277 of which were expressed at extremely low or undetectable levels in all sample types (Supplementary Table).

To further explore the relationships between chromatin accessibility and gene expression, a correlation analysis between these two parameters was performed using only DEGs that were present within DARs. This analysis revealed no significant correlation between gene expression and chromatin accessibility (Figure 5B,C). However, some individual genes did show clear relationships between gene expression and chromatin accessibility. For example, *GNB4* was upregulated and present in a gain-DAR in both F5 and F7 compared to F3 cells (Figure S4A); *FGD5* was downregulated and present in a loss-DAR in both F5 and F7 compared to F3 cells (Figure S4B). In contrast, a significant decrease in *SLIT3* expression in F7 cells was not associated with a significant difference in chromatin accessibility (Figure 5D). Similarly, increases in *ARID5B* and *MFN1* expression in F5 and F7 compared to F3 cells were not associated with any significant changes in chromatin accessibility (Figure 5E,F).

### 3.6. Validation by qRT-PCR

**Figure 6:**
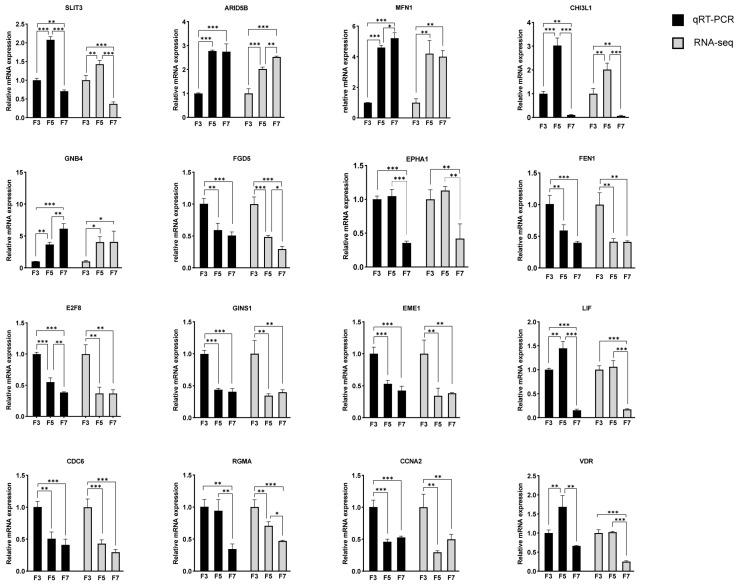
Quantitative reverse transcription PCR (qRT-PCR) validation of RNA sequencing (RNA-seq) data. FPKM, fragments per kilobase of transcript per million mapped reads. Data displayed as mean ± SD, n = 3 independent experiments. * *p* < 0.05, ** *p* < 0.01, *** *p* < 0.001.

The RNA-seq data were validated with qRT-PCR to ensure reliability. Sixteen differentially expressed genes (DEGs) were selected in F5 and/or F7 cells, which were associated with biological processes like cell cycling and DNA replication for validation. Additionally, potential key genes identified through a combined analysis in the previous step were also included. The expression patterns identified from the RNA-seq data were similar to those revealed with qRT-PCR, demonstrating the consistency and reliability of the sequencing data (Figure 6).

**Figure 7:**
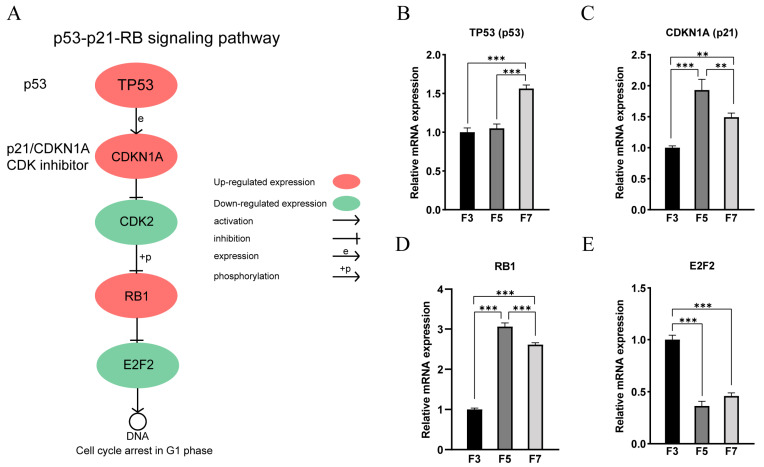
p53-p21-RB signaling pathway. (**A**) Schematic representation of the p53-p21-RB signaling pathway. (**B**–**E**) qRT-PCR results of (**B**) *TP53* (*p53*), (**C**) *CDKN1A* (*p21*), (**D**) *RB1*, (**E**) *E2F2*. ** *p* < 0.01, *** *p* < 0.001.

We observed significant alterations in gene expression levels within the p53-p21-RB signaling pathway, which is involved in cell cycle regulation, as successive cell passages progressed (Figure 7A). Specifically, the expression of P53 exhibited a substantial increase in the F7 cell (Figure 7B). Similarly, the expression of p21 significantly increased in the high cell passages (Figure 7C). The protein encoded by p21 acted as a potent inhibitor of CDKs, leading to a reduction in CDK2 expression. Moreover, a noteworthy upregulation in the expression of RB was also detected in the higher cell passages (Figure 7D). RB interacts with members of the E2F family, creating the RB-E2F complex that suppresses E2F’s transcriptional activity. Consequently, this interaction potentially induces a significant decline in the expression of E2F2 within the higher cell passages (Figure 7E). Although these genes exhibit significant differences in expression levels, there are no significant differences in chromatin accessibility in the gene expression regions. These modifications and regulatory mechanisms in gene expression within the p53-p21-RB signaling pathway likely play a direct role in the G1 phase arrest that we observed in the high cell passages.

## 4. Discussion

Satellite cells are essential for muscle repair functions. One of the primary challenges in long-term culturing of satellite cells derived from livestock is the maintenance of stem cell functionality [15]; skeletal muscle satellite cells that have undergone more than five passages of in vitro expansion exhibit substantially reduced potential for differentiation and lose their ability to repair muscle damage in vivo [17]. To elucidate the underlying molecular mechanisms, we analyzed primary skeletal muscle satellite cells isolated from Landrace pigs in the third, fifth, and seventh passages. In addition to standard cell proliferation assays, a multi-omics analysis approach was implemented, integrating RNA-seq and ATAC-seq data. This approach enabled the examination of alterations in both chromatin accessibility and gene expression during in vitro culturing and correlations of these data with cellular phenotypes. In the process of cell culturing, we found that as the cell passages increased, the rate of EdU staining positivity decreased. This demonstrated a gradual decline in cell proliferative capacity, as is consistent with previous studies [7]. Furthermore, we observed a marked rise in the percentage of cells in the G1 phase as the passage number increased. Existing research indicates that growth arrest in senescent cells may result from blockading of the cell cycle in the G1 phase, which impedes initiation of DNA replication in damaged cells [38,39,40]. Overall, these results indicate that the proliferative capacity of skeletal muscle satellite cells weakened with the passage number and that cells in later passages may have begun senescence.

RNA-seq was conducted to identify genes that may have been involved in changes associated with extended in vitro culturing. Ribosomal RNAs (rRNAs), transfer RNAs (tRNAs), and messenger RNAs (mRNAs) undergo processing, and there is evidence suggesting that the metabolism of all three RNA species is associated with aging and/or cellular senescence [41]. Genes that were downregulated in F5 and/or F7 compared to F3 cells were primarily enriched in the biological processes of RNA processing, rRNA metabolism, RNA binding, and ATP-dependent DNA activity. Reductions in proteostasis, which refers to the balance between protein synthesis, folding, and degradation, promote cellular senescence [10,42]. Genes that were upregulated in F5 and/or F7 compared to F3 cells were primarily enriched in the biological processes of proteolysis involved in protein catabolic processes and modification-dependent protein catabolic processes. In contrast, genes that were upregulated in high-passage cells were significantly enriched in pathways related to apoptosis, including the PI3K-Akt, MAPK, Ras, and p53 signaling pathways. Previous research has demonstrated that genes exhibiting notable variations in aging cells are abundantly present in a range of Gene Ontology (GO) terms, encompassing cell cycle regulation, DNA replication, and DNA repair processes [43,44]. In our research, RNA-seq analysis showed that high-passage cells experienced downregulation of genes associated with DNA replication, DNA repair, cell cycle process, RNA processing, RNA binding, and rRNA metabolism. These transcriptomic phenomena may explain the decline in proliferative capacity observed in high-passage skeletal muscle satellite cells.

ATAC-seq was also conducted to determine whether there were distinct changes in chromatin accessibility across passages. We found that there were fewer regions with significant differences in chromatin accessibility in high-passage cells. In F7 compared to F3 cells, there were only 149 regions of more accessible chromatin (gain-DARs) and 767 regions of less accessible chromatin (loss-DARs). In F5 compared to F3 cells, there were only 198 gain-DARs and 1487 loss-DARs. High-passage cells showed a notable reduction in chromatin accessibility, which occurred most often in intergenic regions; this was consistent with prior studies on aging cells [43]. Interestingly, in comparison to F3 cells, F5 cells showed a greater number of loss-DARs than F7 cells did. Genes with reduced chromatin accessibility in F5 cells were enriched in signaling pathways associated with cell proliferation. Additionally, there was significant enrichment of Atf4 binding sites in loss-DARs among both F7 and F5 compared to F3 cells. We observed a significant enrichment of Atf4 binding sites within the distal intergenic DARsin both F5 and F7 compared to F3 cells. Moreover, DARs located in both the promoter and intron regions displayed a remarkable enrichment of Atf4 binding sites among F5 compared to F3 cells. Although prior studies have reported the involvement of Atf4 in muscle atrophy [45,46,47], a mechanism of action for this transcription factor has not been elucidated in muscle satellite cells. However, a recent study demonstrated that the paralog Atf3 can prevent premature activation of muscle satellite cells [48], suggesting that Atf4 may have an unknown function in these cells. The prevalence of DARs in intergenic regions rather than promoter regions suggested that other gene regulatory elements may have contributed to the alterations in gene expression levels.

We here conducted an integrated transcriptomic–epigenomic analysis to identify relationships between chromatin accessibility and gene expression across passages. There was no general correlation between chromatin accessibility and gene expression levels; DARs were most commonly associated with minimal or no expression changes in the corresponding genes. However, compared to F3 cells, there were 21 and 18 genes in F5 and F7 cells, respectively, with concurrent increases in both chromatin accessibility and gene expression levels. One such gene was *GNB4*, which prior studies have identified as associated with gastric cancer [49]. No previous experiments have linked *GNB4* with skeletal muscle satellite cell functionality, leaving the potential mechanism of action unknown. Furthermore, decreased chromatin accessibility was associated with upregulation of 134 and 69 genes in F5 and F7 cells, respectively, compared to F3 cells. Moreover, compared to F3 cells, there were 14 and 40 genes in F5 and F7 cells, respectively, with concurrent decreases in both chromatin accessibility and gene expression levels. For example, *FGD5*, which regulates PI3 kinase beta in endothelial cells to promote neovascularization [50], was downregulated and present in gain-DARs in F5 and F7 cells compared to F3 cells. Similar to *GNB4*, a potential mechanism of *FDG5* action in skeletal muscle satellite cells is currently unknown. Thus, both *GNB4* and *FGD5* may have undiscovered mechanisms of action in muscle satellite cells.

Notably, *SLIT3* was significantly downregulated in F7 cells, despite a lack of significant differences in chromatin accessibility. Previous studies have implicated *SLIT3* in myogenic differentiation, and mice with *SLIT3* defects have reduced skeletal muscle mass, muscle strength, and physical activity levels [51]. In contrast, *ARID5B* and *MFN1* were significantly upregulated in higher-passage cells without any noticeable variation in chromatin accessibility. Prior research has shown that *arid5b* primary skeletal muscle cells exhibit deficiencies in differentiation and sarcomere assembly [52], and that *MFN1* interacts with *miR-142a-5p* to regulate denervation-induced skeletal muscle atrophy via modulation of mitochondrial dysfunction, mitophagy, and apoptosis [53]. Overall, these observations suggest that *SLIT3*, *ARID5B*, and *MFN1* may underlie the observed decreases in proliferative capacity and increased senescence among skeletal muscle satellite cells following extended in vitro culture. Further functional studies will be necessary to validate these results.

Using qRT-PCR, we further validated significant differences in the expression levels of genes associated with cell cycling and DNA replication among cells at different passages. For instance, genes such as *FEN1*, *GINS1*, *Eme1*, *CDC6*, and *CCNA2* showed significantly decreased expression in high passage cells. FEN1 (Flap Endonuclease 1) is an enzyme that plays a crucial role in DNA replication and repair processes. It facilitates the maturation of Okazaki fragments by cleaving the RNA-DNA flap structure, which is a necessary step for joining these fragments into a continuous DNA chain [54]. GINS1 is a subunit of the GINS complex, which plays a crucial role in DNA replication in eukaryotes, and its participation in DNA replication is closely associated with cell cycle regulation [55]. Eme1 is a component of the Mus81-Eme1 complex, which is activated in response to DNA damage and plays a critical role in DNA replication, repair, and recombination processes [56]. *CDC6* plays a crucial role in DNA replication as it is involved in the recognition of replication origins, recruitment and loading of helicase, and activation of replication origins. The CDC6 protein interacts with the Origin Recognition Complex (ORC) to form the ORC-CDC6 complex. With the assistance of Cdt1 (Cdc10-dependent protein 1), the core Mcm2-7 helicase is loaded onto the replication origins of DNA through a series of steps [57]. CCNA2 (Cyclin A2) plays a significant role in the cellular senescence process. It acts as a crucial regulatory factor during the G1/S transition in the cell cycle by binding to and activating Cdk2. Silencing *CCNA2* (i.e., inhibiting gene expression) markedly enhances cell senescence, whereas overexpression of *CCNA2* can effectively delay cell senescence and reverse the aging effects induced by miR-124 and miR-29 [58].The expression levels of these genes significantly decrease in high-passage cells, which may be one of the reasons contributing to weakened cell proliferative ability and cell cycle arrest.

The p53-p21-RB signaling pathway is a complex network that regulates cellular processes and involves three key proteins: the tumor suppressor TP53 (p53), the cyclin-dependent kinase inhibitor CDKN1A (p21), and the retinoblastoma protein RB. This pathway is critical in responding to DNA damage and cellular stress by controlling the cell cycle and preventing unrestricted proliferation of damaged cells, thus playing a crucial role in preventing cancer development [59]. p53, as a transcription factor, is normally present at low levels but becomes more stable and active in response to DNA damage and stress. p21 is a direct target of p53 and encodes a potent inhibitor of CDKs, which can bind to multiple CDKs, including CDK1, CDK2, CDK4, and CDK6, thereby inhibiting their activity. This inhibition leads to the arrest of the cell cycle, particularly in the transition from G1 to S phase. RB protein is essential for transcriptional regulation throughout the cell cycle. During the G1 phase, unphosphorylated RB binds to E2F family transcription factors, forming the RB-E2F complex, which suppresses the transcriptional activity of E2F and the expression of E2F-dependent cell cycle genes involved in DNA replication and cell division. As the cell cycle progresses, RB is phosphorylated by CDKs, resulting in dissociation of the RB-E2F complex and release of E2F. This allows for the expression of cell cycle genes and progression into the S phase [60]. In our study, we observed an upregulation of p53 gene expression in high-grade cells, resulting in increased levels of p21 gene. Subsequently, the elevated p21 gene acted as an inhibitor of CDKs, particularly CDK2, leading to the dephosphorylation of RB. This dephosphorylation facilitated the formation of the RB-E2F complex by promoting its binding with E2F family members, with a specific emphasis on E2F2. As a consequence, the transcriptional activity of E2F and the expression of E2F-dependent cell cycle genes were suppressed. Although there are notable disparities in the expression levels of these genes, no significant differences are detected in chromatin accessibility within the gene expression regions. We propose that the regulation of the p53-p21-RB signaling pathway may contribute to G1 phase arrest in high-grade cells.

## 5. Conclusions

High-passage porcine skeletal muscle satellite cells clearly showed significant weakening of the proliferative capacity and extensive alterations in the expression levels of genes associated with proliferation. Additionally, the cells exhibited G1 phase arrest, the p53-p21-RB signaling pathway may play a crucial regulatory role in these processes. The lack of a correlation between chromatin accessibility and gene expression indicated that chromatin accessibility may not have directly altered the promoter region activity of DEGs in high-passage cells. *GNB4*, *FGD5*, *SLIT3*, *ARID5B*, and *MFN1* were identified as putative key genes to the decreased proliferative capacity of high-passage cells. Moreover, the p53-p21-RB signaling pathway may play a crucial regulatory role in cell proliferation processes. This study serves as a valuable reference for future research into the in vitro cultivation of skeletal muscle satellite cells, promoting viable muscle cell culturing methods.

## Data Availability

ATAC-seq and RNA-seq data were deposited in CNCB (China National Center for Bioinformation) with an identifier: PRJCA023066.

## Supplementary Materials

The following supporting information can be downloaded at: https://www.mdpi.com/article/10.3390/cells13121031/s1, Figure S1: Immunofluorescence staining of skeletal muscle satellite cells and Negative Control; Figure S2: Gene expression pattern analysis; Figure S3: Differentially accessible chromatin region (DAR) analyses; Figure S4: Genes with significant differences in both chromatin accessibility and expression levels; Table S1: Quantitative reverse transcription PCR primer sequences; Table S2: RNA sequencing data statistics; Table S3 Transposase-accessible chromatin sequencing data statistics.
