# Supplementary material for: Integrated ATAC-seq and RNA-seq Analysis of In Vitro Cultured Skeletal Muscle Satellite Cells to Understand Changes in Cell Proliferation
Source: Cells. 2024 Jun 13;13(12):1031. doi: 10.3390/cells13121031 (PMC11201436; doi:10.3390/cells13121031)
Supplement: Supplementary file 1 [file cells-13-01031-s001.zip › cells-3051520-supplementary.pdf]

Integrated ATAC-seq and RNA-seq Analysis of In Vitro Cultured Skeletal Muscle Satellite Cells to Understand Changes in Cell Proliferation

Zeyu Ren [1,2,†], Siyi Zhang [1,2,†], Liangyu Shi [1], Ao Zhou [1], Xin Lin [3], Jing Zhang [1,*,‡], Xiusheng Zhu [2,*,‡], Lei Huang [2,*,‡] and Kui Li [2]

Contains:

Figure S1 Immunofluorescence staining of skeletal muscle satellite cells and Negative Control.

Figure S2 Gene expression pattern analysis.

Figure S3 Differentially accessible chromatin region (DAR) analyses.

Figure S4 Genes with significant differences in both chromatin accessibility and expression levels.

Table S1 Quantitative reverse transcription PCR primer sequences.

Table S2 RNA sequencing data statistics.

Table S3 Transposase-accessible chromatin sequencing data statistics.

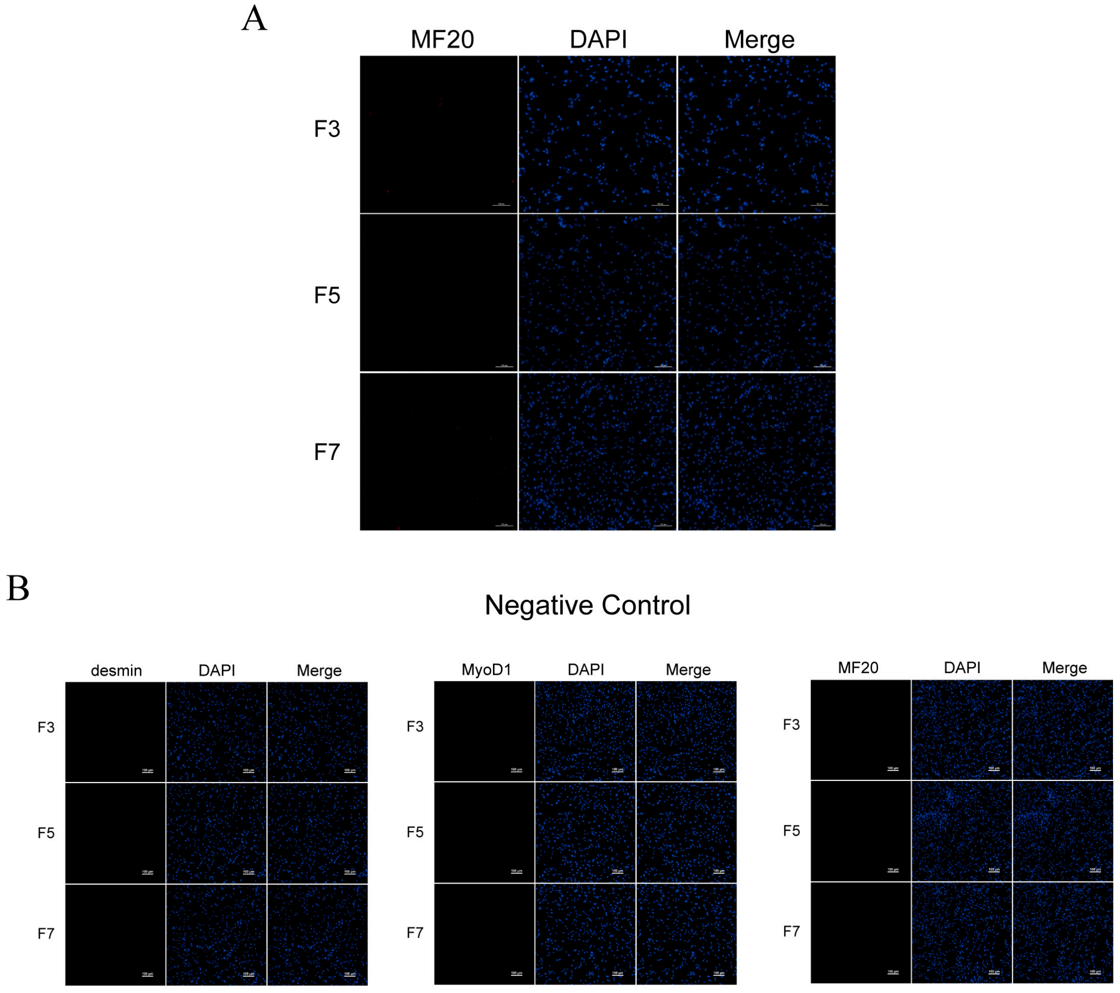

Figure S1. Immunofluorescence staining of skeletal muscle satellite cells and Negative Control. **(A)** MF20 immunofluorescence staining on F3, F5

and F7 passages of skeletal muscle cells at a magnification of 200×. **(B)** Negative controls for desmin, MyoD1 and MF20. The negative control group was established by omitting the addition of primary antibody.

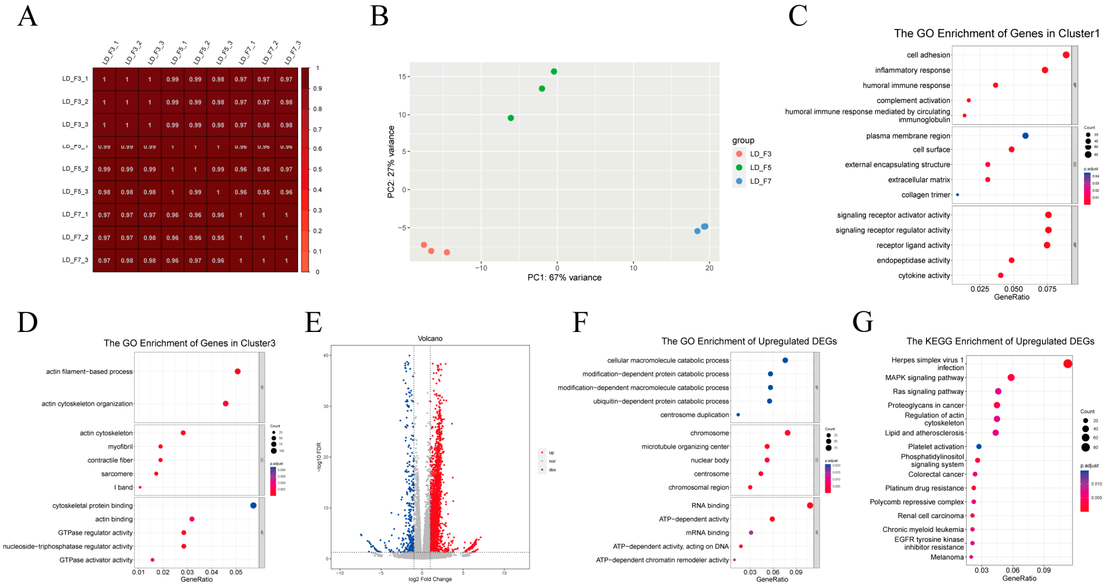

Figure S2. Gene expression pattern analysis. **(A)** Pairwise Pearson correlation analysis for replicate samples of third-passage (F3), fifth-passage (F5), and seventh-passage (F7) pig skeletal muscle cells. **(B)** Principal component analysis of all nine samples. **(C, D)** Gene Ontology (GO) enrichment analysis of the genes in clusters **(C)** 1 and **(D)** 3. **(E)** Fold-change values for each gene in F5 compared to F3 cells. Gray points were not statistically significantly different between samples; red and blue points were significantly upregulated and downregulated, respectively, in F5 compared to F3 cells. Differences were considered statistically significant at |log2(fold change) ≥ 1| and false discovery rate-adjusted $p < 0.05$. **(F, G)** Functional enrichment analyses of upregulated DEGs in F5 VS F3 gruop with **(F)** Gene Ontology (GO) annotations and **(G)** Kyoto Encyclopedia of Genes and Genomes (KEGG) biochemical pathway annotations.

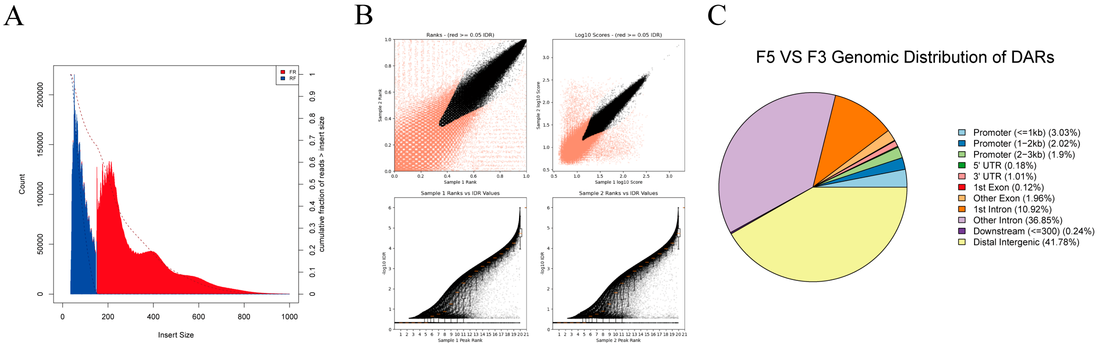

D

F5 VS F3 Loss DARs in Distal Intergenic

Name	Motif	P-value
Atf4	ATGATGCAAT	1×10^{-178}
RUNX	TGTGGTTA	1×10^{-30}
TEAD4	CCTGGAATGT	1×10^{-27}
Fosl2	GGATGAGTCA	1×10^{-26}
NF1-halfsite(CTF)	TTTGGCAAGA	1×10^{-20}

F7 VS F3 Loss DARs in Distal Intergenic

Name	Motif	P-value
Atf4	ATTGCATCAG	1×10^{-137}
Fos	TGAGTCAC	1×10^{-41}
TEAD4	ACATTCCA	1×10^{-19}
ASH1	AGTCAGCC	1×10^{-14}
HIC1	CTGGCAGACG	1×10^{-13}

E

F5 VS F3 Loss DARs in Promoter Reigons

Name	Motif	P-value
Atf4	ATGATGCAAT	1×10^{-43}
Chop	ATTGCATCAT	1×10^{-40}
CEBP:AP1	GATGTTGCAA	1×10^{-37}
AARE	GATTGCATCA	1×10^{-27}
HLF	GTTATGTAAC	1×10^{-27}

F5 VS F3 Loss DARs in Intron Reigons

Name	Motif	P-value
Atf4	ATGATGCAAT	1×10^{-231}
RUNX2	TATGTGGTTA	1×10^{-34}
TEAD3	TGGAATGC	1×10^{-24}
BATF3	TGAGTAAT	1×10^{-19}
Bcl11a	CTGGCCAGGC	1×10^{-17}

Figure S3. Differentially accessible chromatin region (DAR) analyses. **(A)** Distribution of insert fragment sizes. **(B)** Concordance plot showing peaks between replicate samples as evaluated with Irreproducibility Discovery Rate (IDR). Top left, the ranks of peaks in Rep1 compared to Rep2; top right, log10(peak scores) in Rep1 compared to Rep2. Peaks that failed to pass the IDR threshold shown in red. Bottom left and right, relationships between peak ranks and IDR scores. Boxplots represent the distribution of IDR values with a default threshold of -1×10^{-6}**(C)** Genomic distribution of DARs in F5 VS F3 gruop. **(D)** Enriched transcription factor binding motifs in F5 and F7 compared to F3 cells in DARs located in distal intergenic. **(E)** Enriched transcription factor binding motifs in F5 VS F3 gruop in DARs located in promoter and intron reigons.

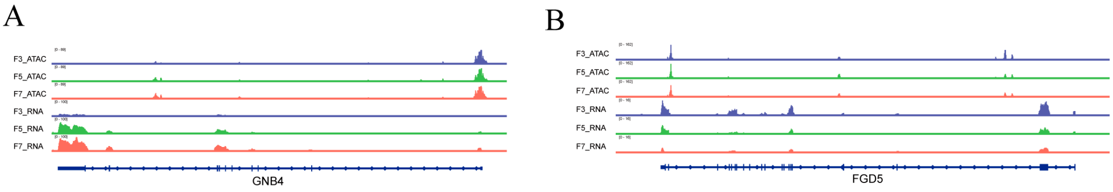

Figure S4. Genes with significant differences in both chromatin accessibility and expression levels. **(A)** Integrative Genomics Viewer (IGV) snapshot of *GNB4*, for which both chromatin accessibility and gene expression levels were increased in fifth-passage (F5) and seventh-passage (F7) compared to third-passage (F3) cells. **(B)** IGV snapshot of *FGD5*, for which both chromatin accessibility and gene expression levels were decreased in F5 and F7 cells.

Table S1. Quantitative reverse transcription PCR primer sequences.

Number	Name	Sequence (5′ to 3′)
1	ARID5B_F	GTACTTACCTTATCTAGGCTGT
2	ARID5B_R	AGCCCACAAATATGTCCTC
3	CCNA2_F	ACCTGGACCAAGAAAACCACT
4	CCNA2_R	TGTCCTCATGGTAGTCTGGAAC
5	CDC6_F	CCGACAATTAAGTCTCCCAGCAA
6	CDC6_R	CTTGAACAATCGCATACGAGCAG
7	CHI3L1_F	ACGCTTTCCTCTCACCAGTG
8	CHI3L1_R	GGTACTCCGACTCCTAGCCA
9	E2F8_F	TGCACAAAGAAAACGCTGTGA
10	E2F8_R	ATACTGCAACCACAACGGAT
11	EME1_F	TGCACAAAGAAAACGCTGTGA
12	EME1_R	ATACTGCAACCACAACGGAT
13	EPHA1_F	CTGCGGACCATTGCCAATTT
14	EPHA1_R	GGAAGTGCAGGATGTAGCGT
15	FEN1_F	CCCGTCGTTACCTGATCTCG
16	FEN1_R	AACTGGCTGCCCTCACTTTT
17	FGD5_F	CGCCTCTTCCAGTACCAAGTGC
18	FGD5_R	CCCTTGCTCCATGCTGTCGTTG
19	GINS1_F	CACTCAGGTGGGAGTATGGCC
20	GINS1_R	TGTGTGATGTCCAAGCCTTCA
21	GNB4_F	TTGTGCTTATGCTCCCTCTGGT
22	GNB4_R	GGCTCACTCTCACATTTCCCTC
23	LIF_F	CCGCCTGTGCAACAAGTACCA
24	LIF_R	ACAGAGATGACCTGCTTATACTTCC
25	MFN1_F	TGGACTTTATCCGAAACCAGA
26	MFN1_R	CATGAAACCTTATTTGCCACCT
27	RGMA_F	AGTTGTCTCAGCCCTTAGCATC
28	RGMA_R	AGCCTGGTTGAAAGTCTCCT
29	SLIT3_F	CATCGTCGAAATACGCCTGGAA
30	SLIT3_R	CTTGCTGATGTCTATTCGCTTC
31	VDR_F	TTCCTTACCTGACCCCGGCGACT
32	VDR_R	CCTTGCGCTTCATGCTCCGTCT
33	GAPDH_F	ATTGTCGCCATCAATGACCC
34	GAPDH_R	TGGCCTTTCCATTGATGACAAGC
35	RB1_F	ACTTGGAGTCCGATTGTATTACCG
36	RB1_R	CCATTACAACCTCAAGAGCGCAT
37	E2F2_F	AGCTTGTGTCCCCTTCTTACCG
38	E2F2_R	AATTGGCTTTTCTTCTACCTGGAT
39	TP53_F	ACCCTCAACTAATATGACGGAT
40	TP53_R	CTTCATCTACATGCACATCTCG
41	CDKN1A_F	TCAACGCCTGGAACTACTCCC
42	CDKN1A_R	CCTAGCCACAGCCTCTACTCC

Table S2. RNA sequencing data statistics.

Sample ID#	Raw data	Clean data*	Mapping rate (%)
LD_F3_1	43,123,232	39,955,217	92.65
LD_F3_2	42,695,442	39,673,714	92.92
LD_F3_3	47,236,435	43,915,924	92.97
LD_F5_1	35,252,133	32,811,147	93.08
LD_F5_2	36,212,985	33,661,386	92.95
LD_F5_3	65,616,856	59,534,390	90.73
LD_F7_1	41,868,785	38,731,481	92.51
LD_F7_2	52,083,909	48,747,417	93.59
LD_F7_3	49,284,000	46,144,208	93.63

#Sample ID demonstrates the following basic information: LD (Landrace skeletal muscle satellite cells), F3 (third-passage), F5 (fifth-passage), F7 (seventh-passage).

*Clean data: reads were mapped to S. scrofa v11.1 reference genome (susScr11)

Table S3. Transposase-accessible chromatin sequencing data statistics.

Sample ID#	Raw data	Mapped rate (%)*	Clean data&	Peaks number**	FRIP value***
LD_F3_1	136,462,336	97.43%	97,319,898	149,320	0.65
LD_F3_2	109,550,884	97.60%	80,021,602	147,921	0.65
LD_F3_3	139,283,554	97.33%	97,730,648	151,414	0.63
LD_F5_1	96,853,782	97.68%	71,761,718	148,904	0.68
LD_F5_2	120,508,692	97.49%	85,766,210	150,252	0.65
LD_F5_3	124,669,856	97.43%	88,337,558	148,544	0.64
LD_F7_1	90,506,760	97.22%	60,201,410	135,813	0.54
LD_F7_2	52,648,576	97.20%	36,232,316	124,469	0.56
LD_F7_3	71,532,622	97.17%	47,911,930	133,694	0.53

#Sample ID demonstrates the following basic information: LD (Landrace skeletal muscle satellite cells), F3 (third-passage), F5 (fifth-passage), F7 (seventh-passages).

*Mapped rate: The proportion of data mapped to the genome after alignment.

&Clean data: Reads after unmapped reads, mitochondrial DNA, and duplicates removed

**Peaks number: The number of peaks called by MACS2

***FRIP, fraction of reads in called peak regions
